# Responses of nitrogen metabolism and seed nutrition to drought stress in soybean genotypes differing in slow-wilting phenotype^1^

**DOI:** 10.3389/fpls.2013.00498

**Published:** 2013-12-10

**Authors:** Nacer Bellaloui, Anne M. Gillen, Alemu Mengistu, Hirut Kebede, Daniel K. Fisher, James R. Smith, Krishna N. Reddy

**Affiliations:** ^1^Crop Genetics Research Unit, US Department of Agriculture - Agricultural Research ServiceStoneville, MS, USA; ^2^Crop Genetics Research Unit, US Department of Agriculture - Agricultural Research ServiceJackson, TN, USA; ^3^Crop Production Systems Research Unit, US Department of Agriculture - Agricultural Research ServiceStoneville, MS, USA

**Keywords:** soybean, seed nutrition, seed composition, slow-wilting, drought tolerance

## Abstract

Recent advances in soybean breeding have resulted in genotypes that express the slow-wilting phenotype (trait) under drought stress conditions. The physiological mechanisms of this trait remain unknown due to the complexity of trait × environment interactions. The objective of this research was to investigate nitrogen metabolism and leaf and seed nutrients composition of the slow-wilting soybean genotypes under drought stress conditions. A repeated greenhouse experiment was conducted using check genotypes: NC-Roy (fast wilting), Boggs (intermediate in wilting); and NTCPR94-5157 and N04-9646 (slow-wilting, SLW) genotypes. Plants were either well-watered or drought stressed. Results showed that under well-watered conditions, nitrogen fixation (NF), nitrogen assimilation (NA), and leaf and seed composition differed between genotypes. Under drought stress, NF and NA were higher in NTCPR94-5157 and N04-9646 than in NC-Roy and Boggs. Under severe water stress, however, NA was low in all genotypes. Leaf water potential was significantly lower in checks (−2.00 MPa) than in the SLW genotypes (−1.68 MPa). Leaf and seed concentrations of K, P, Ca, Cu, Na, B were higher in SLW genotypes than in the checks under drought stress conditions. Seed protein, oleic acid, and sugars were higher in SLW genotypes, and oil, linoleic and linolenic acids were lower in SLW genotypes. This research demonstrated that K, P, Ca, Cu, Na, and B may be involved in SLW trait by maintaining homeostasis and osmotic regulation. Maintaining higher leaf water potential in NTCPR94-5157 and N04-9646 under drought stress could be a possible water conservation mechanism to maintain leaf turgor pressure. The increase in osmoregulators such as minerals, raffinose, and stachyose, and oleic acid could be beneficial for soybean breeders in selecting for drought stress tolerance.

## Introduction

Soybean is a major crop in the world and a source of protein, oil, sugars, and minerals. Water deficit is an important environmental stress factor that negatively impacts crop growth and development, leading to yield loss and poor seed quality (Smith et al., 2008; Mengistu et al., 2010; Bellaloui et al., 2012; Furlan et al., 2012). Water deficit triggers physiological and biochemical responses such as: (a) cellular dehydration, causing osmotic stress and removal of water from the cytoplasm into the extracellular space, lowering turgor pressure and cytosolic and vacuolar volumes (Bartels and Sunkar, 2005); (b) accumulation of compatible solutes such as sugars (sucrose, hexoses, and sugar alcohols such as mannitol, sorbitol, and isotol) (Gilmour et al., 2000 Streeter et al., 2001; Taji et al., 2002), amino acids such as proline, glycine, and betaine to maintain turgor and stabilize proteins and cell structures (Yancey et al., 1982; Burg et al., 1996); (c) accumulation of specific ions such as K to increase osmotic pressure and regulate stomatal opening (Bartels and Sunkar, 2005); (d) expression of stress-induced genes (Bartels and Sunkar, 2005); (e) increases in hormones such as abscisic acid; and (f) stimulation of signaling components such as protein kinases and phosphatases, Ca^2+^, Cl^−^, Na^+^, K^+^, and reactive oxygen species (ROS) (Knight et al., 1997; Schroeder et al., 2001; Furlan et al., 2012).

Nitrogen fixation and assimilation are negatively affected by drought. Nitrogen metabolism in legumes is a result of both symbiotic N_2_ fixation and mineral N assimilation processes. Atmospheric N_2_ is fixed by the enzyme nitrogenase in the bacteroids of nodules (Kanayama et al., 1999), and nitrate reduction (assimilation) (NR) is catalyzed by the enzyme nitrate reductase (NR). Both NR and nitrogenase enzymes coexist in nodules competing for reductant (reducing power) (Caba et al., 1995). Drought affects both nitrogen fixation (NF) and nitrogen assimilation (NA) by inhibiting nitrogenase and nitrate reductase, although nitrogenase is more sensitive to drought stress than nitrate reductase (Purcell and King, 1996). Mechanisms involved in the physiological response of nitrogen fixation to drought have been previously reported and included carbon shortage, nodule carbon metabolism, oxygen limitation, and feedback of nitrogen fixation products (Serraj et al., 1999; Serraj, 2003) such as amides (mainly asparagine) and ureides (allantoin and allantoic acid), both exported to the shoot via the xylem (Schubert et al., 1995).

Mineral nutrition in plants is essential for plant growth, development, production, and seed quality. Deficiencies in minerals due to abiotic stress such as drought at any plant stage result in yield loss and poor seed quality. Physiological and biochemical roles of macro-nutrients such as S, Ca, K, Mg, and P or micronutrients such as Fe, B, and Zn have been previously reported for plants (Mengel and Kirkby, 1982; Marschner, 2012). However, the physiological and biochemical roles of mineral nutrients in slow-wilting genotypes under drought tolerance have not been well investigated.

Recent advances in soybean breeding resulted in selection of slow-wilting soybean genotypes with drought tolerance. Slow-wilting is defined as a delayed wilting response to decreased soil moisture as compared to an average soybean cultivar. However, mechanisms involved in plant responses to drought stress are not well understood (Lawlar and Cornic, 2002; Hufstetler et al., 2007; Charlson et al., 2009; King et al., 2009). Evidence showed that the wilting response (fast or slow/delayed wilting) of soybean genotypes to drought stress differ (Sloane et al., 1990), and slow-wilting genotypes have the ability to conserve soil moisture (Fletcher et al., 2007; King et al., 2009; Ries et al., 2012) by lowering transpiration rate to maintain turgor pressure compared with conventional fast-wilting genotypes (Charlson et al., 2009). King et al. (2009) reported that volumetric soil water content was greater for slow-wilting than for fast-wilting genotypes in a field experiment, but wilting responded similarly for both genotypes. In a growth chamber experiment, transpiration declined similarly in response to drought stress for fast- and slow-wilting genotypes, indicating that more than one mechanism is involved in slow-wilting (King et al., 2009). Also, Sloane et al. (1990), using a slow-wilting genotype PI 416937 and “fast-wilting” cultivar Forrest, found that water stress reduced leaf water potential equally for both genotypes, but that PI 416937 maintained lower levels of solute potential and higher pressure potential and relative water content than Forrest. They suggested that under water stress PI 416937 may accumulate more solutes in leaves than Forrest, and concluded that PI 416937 may be an important source of drought tolerance for breeding programs.

In spite of the extensive research on drought tolerance, identification of physiological and biochemical traits involved with the slow-wilting trait has not been fully explored, and may involve several mechanisms (King et al., 2009). Therefore, the objective of this research was to evaluate nitrogen metabolism (nitrogen fixation and nitrate assimilation) and identify possible roles of sugars and minerals in unique genotypes that have a slow-wilting trait. Our hypothesis was that if drought tolerance in the slow-wilting genotypes leads to conserving soil moisture under drought tolerance, then leaf and seed nutrients involved in osmoregulation components would respond differently from those grown under well-watered conditions.

## Materials and methods

### Growth conditions

A repeated greenhouse experiment was conducted at USDA-ARS, Crop Genetics Research Unit. Soybean genotypes in maturity group (MG) VI and differing in wilting were used. Genotypes were: NC-Roy (fast wilting), Boggs (intermediate in wilting), and slow-wilting (SLW) genotypes: NTCPR94-5157 and N04-9646. Soybean seeds were germinated in flat trays in vermiculite. Uniform size seedlings at about the V1 stage (fully developed unifoliate leaves and one unrolled trifoliate leaf) were transplanted into 9.45 L pots filled with field soil. Soil characteristics were silty loam with pH 6.3, 1.1% organic matter, a cation exchange capacity of 15 cmol/kg, and soil textural fractions of 26% sand, 56% silt, and 18% clay. The soil contained an abundant native population of *B. japonicum*. Soil in pots, weighed, saturated with deionized water, and drained. The pots were then weighed to obtain the water field capacity using Watermark 200SS soil sensors inserted in the pots and read daily with a Soil Moisture Meter (Irrometer Company, Inc., Riverside, California, USA). Well watered plants were kept between −15 and −20 kPa (this was considered field capacity for the control plants), and drought stressed plants were kept between −90 and −100 kPa (Bellaloui et al., 2010a). Plants were considered fully matured when they reached R8 (full maturity) according to Fehr and Caviness (1977). At full maturity, 95% of pods reached full maturity). Three replicates were used in each treatment, and experiment was repeated. Therefore, the total replicates used in this experiment is 6. Each pot had three plants. Greenhouse temperature conditions were maintained at 34 ± 9°C during the day and 28 ± 7°C at night. Photosynthetic photon flux density (PPFD) during the day of about 800-2300 μmol·m^−2^·s^−1^ was measured by a Quantum Meter (Spectrum Technologies, Inc., Aurora. Illinois, USA). The wide range of light intensity reflects a bright, sunny, or cloudy day. The two experiments were conducted simultaneously in two different bays during the normal growing season (from April to September) to simulate the growing season photoperiod of soybean production in the midsouth USA. The fully expanded leaves at seed-fill stages (R5–R6) were analyzed for NF, NA, and mineral nutrition. Seed fill stage was chosen as it is considered as the most critical stage for soybean for seed evaluation, and any impact on nutrients uptake or movement from leaves to seed will impact seed quality. Mature seeds at R8 were harvested and analyzed for seed protein, oil, fatty acids, sugars, and minerals. In a separate preliminary experiment to further investigate the role of K and B (among the responsive nutrients to SLW trait under drought), a foliar K application at a rate of 1.75% K_2_SO_4_ and foliar B application at a rate of 1.1 kg ha^−1^ H_3_BO_3_ were made at seed-fill stages (R5–R6). Leaves were collected for K and B analysis 2 weeks after K and B application. Foliar K and B were applied only to SLW genotypes under moderate drought stress (−99 to −100 kPa) and severe drought stress (soil water potential = −199 kPa).

### Minerals, N, and S analyses in leaves and seeds

The fully expanded youngest leaves at seed-fill stage (R5–R6) were collected from each treatment and replicate and were analyzed for macro- and micronutrients. Mature seeds at R8 were collected and analyzed for seed minerals, and N and S concentrations. Leaf and seed samples were ground to pass through a 1-mm sieve using a Laboratory Mill 3600 (Perten, Springfield, IL). Leaf and seed macro- and micro-nutrients were analyzed by digesting 0.5 g of dried ground seed in HNO_3_ in a microwave digestion system. The concentrations of nutrients were determined using inductively coupled plasma spectrometry (ICP) (Bellaloui et al., 2011, 2013). Nitrogen and S were measured in a 0.25-g sample using a LECO CNS-2000 elemental analyzer (LECO Corporation, St. Joseph, MI, USA) (Bellaloui et al., 2011). Concentrations of B, Fe, and P were determined as indicated in the following sections.

### Boron measurement

Boron concentration was determined in fully expanded leaves at seed-fill stages (R5-R6) using the Azomethine-H method (Lohse, 1982; Dordas, 2006; Dordas et al., 2007). Briefly, a 1.0-g seed sample was ashed at 500°C and then extracted with 20 ml of 2 M HCl at 90°C for 10 min and filtered. The filtered mixture was transferred to plastic vials, and a 2-ml sample of the solution was added to 4 ml of buffer solution (containing 25% ammonium acetate, 1.5% EDTA, and 12.5% acetic acid) and 4 ml of freshly prepared azomethine-H solution (0.45% azomethine-H and 1% of ascorbic acid) (John et al., 1975). The concentration of boron in leaves and seeds was determined in the samples after color development at 420 nm using a Beckman Coulter DU 800 spectrophotometer (Beckman Coulter, Inc., Brea, CA, USA).

### Iron measurement

Iron concentration in leaves and seed was measured after acid wet digestion, extraction, and reaction of the reduced ferrous Fe with 1,10-phenanthroline according to the methods of Bandemer and Schaible (1944) and Loeppert and Inskeep (1996). A sample of 2 g of dried ground seed was acid digested (Analytical Methods Committee, 1959). Then, the acids were removed by volatilization, and the soluble constituents were dissolved in 2 M of HCl. Standard solutions of Fe ions were prepared in 0.4 M HCl, and ranged from 0.0 to 4 μg mL^−1^ of Fe. A phenanthroline solution of 0.25% (w/v) was prepared in 25% (v/v) ethanol. The quinol solution (1% w/v) reagent was prepared on the day of use. About 4 mL of the sample solution was added to a 25-mL volumetric flask. The aliquot was diluted to 5 mL using 0.4 M HCl. Quinol solution (1 mL) was added and mixed, and then 3 mL of the phenanthroline solution and 5 mL of the tri-sodium citrate solution (8% w/v) were added. The solution was diluted to 25 mL with distilled water and incubated at room temperature for 4 h. The concentrations of Fe in samples were read at 510 nm using the Beckman Coulter DU 800 spectrophotometer.

### Phosphorus measurement

Concentration of P was measured in leaves at seed-fill stages (R5–R6) and in seeds at maturity (R8). Phosphorus measurement was carried out spectrophotometrically as the yellow phosphor-vanado-molybdate complex according to Cavell (1955). A dried ground leaf and seed sample of 2 g was ashed to completely destroy organic matter. After ashing, 10 mL of 6 M HCl was added. The sample was placed in a water bath to evaporate the solution to dryness, and then kept under heat and 2 mL of 36% v/v HCl was added and the sample was boiled. A volume of 10 mL distilled water was added, and the solution was then boiled for a few seconds, transferred to a 50-mL volumetric flask, diluted to 50 mL with distilled water, and filtered. A volume of 5 mL of 5 M HCl and 5 mL of ammonium molybdate–ammonium metavanadate reagent was added to 5 mL of the filtrate. The solution was diluted with distilled water to 50 mL and allowed to stand for 30 min. Ammonium molybdate–ammonium metavanadate was made by dissolving 25 g of ammonium molybdate and 1.25 g of ammonium metavanadate in 500 mL of distilled water. Phosphorus concentration was measured after color development using the Beckman Coulter DU 800 spectrophotometer at 400 nm. Standards of P solutions with concentrations ranging from 0–50 μg mL^−1^ of P) were prepared using dihydrogen orthophosphates.

### Seed analysis for protein, oil, and fatty acids

Mature seeds were collected from each treatment and replicate and analyzed for protein, oil, and fatty acids. A sample of 25 g of seed was ground using the Laboratory Mill 3600. Analyses were conducted by near infrared reflectance (NIR) (Wilcox and Shibles, 2001; Bellaloui et al., 2009) using a diode array feed analyzer AD 7200 (Perten, Springfield, IL, USA). The initial calibrations were developed by the University of Minnesota using Perten's Thermo Galactic Grams PLS IQ software. The calibration curve was established according to AOAC methods (AOAC, 1990a,b). Analyses of protein and oil were performed based on a seed dry matter basis (Wilcox and Shibles, 2001; Boydak et al., 2002), and fatty acids were analyzed on an oil basis.

### Seed analysis for sucrose, raffinose, and stachyose

Seed at harvest maturity were collected and analyzed for sucrose, raffinose, and stachyose concentrations. A sample of 25 g of seed from each plot was ground using the Laboratory Mill 3600. Analyses were conducted by NIR (Wilcox and Shibles, 2001; Bellaloui et al., 2010b) using the AD 7200 array feed analyzer. Analyses of sugars were performed based on a seed dry matter basis (Wilcox and Shibles, 2001; Boydak et al., 2002).

### Seed glucose determination

Concentration in mature seeds was determined according to an enzymatic reaction using a Glucose (HK) Assay Kit, Product Code GAHK-20 (Sigma-Aldrich Co, St Louis, MO, USA). Glucose during this reaction is phosphorylated by adenosine triphosphate (ATP) in a reaction catalyzed by hexokinase. The glucose-6-phosphate (G6P) produced is then oxidized to 6-phosphogluconate by oxidized nicotinamide adenine dinucleotide (NAD) in a reaction catalyzed by glucose-6-phosphate dehydrogenase (G6PDH). An equimolar amount of NAD is then reduced to NADH, and the increase in absorbance at 340 nm is directly proportional to glucose concentration in the sample. The Glucose (HK) Assay Reagent was reconstituted according to the manufacturers' instructions in 20 ml deionized water. Seed samples were ground using the Laboratory Mill 3600 to obtain uniform particles. A random ground sample of 0.1 mg was extracted with deionized water. Then, the sample solution was heated by heat plate to aid extraction. The extract was diluted to 1:100 with deionized water to obtain a range of 0.05 to 5 mg glucose ml^−1^. A 100 μ l sample was added to 1ml of the Glucose (HK) Assay Reagent and incubated at room temperature for 15 min. A sample blank consisting of 100 μ l of sample and 1ml of deionized water, and a reagent blank consisting of 1ml of Glucose (HK) Assay Reagent and 100μ l of deionized water were also prepared. The absorbance was read at 340 nm using the Beckman Coulter DU 800 spectrophotometer. The concentration of the glucose was expressed as mg g^−1^ dwt.

### Seed fructose determination

The concentration of fructose in seed was determined enzymatically using a Fructose Assay Kit, Product Code FA-20 (Sigma-Aldrich Co., St. Louis, MO, USA). Fructose in this reaction is phosphorylated by ATP in a reaction catalyzed by hexokinase. Fructose 6-phosphate is converted to G6P by phosphoglucose isomerase (PGI). Then, oxidation of G6P to 6-phosphogluconate occurred in the presence of NAD in the reaction catalyzed by glucose-6-phosphate dehydrogenase (G6PDH). An equimolar amount of NAD is then reduced to NADH, and the increase in absorbance at 340 nm is directly proportional to fructose concentration in a sample. Seed samples were ground using the Laboratory Mill 3600 as described above. A random sample of 0.1 mg was extracted with deionized water. The sample solution was heated by heat plate to aid extraction and diluted to 1:100 with deionized water to obtain a range of 100–1000 μg fructose ml^−1^. A sample of 100 μ l was added to 2 ml of the Glucose Assay Reagent and 0.02 ml PGI and incubated at room temperature for 15 min. A sample blank consisting of 100 μ l of sample and 0.02 ml deionized water was prepared, and a sample of Glucose Assay Reagent blank and PGI blank was also prepared as recommended by the manufacturer. Samples were read after 15 min at absorbance 340 nm using the Beckman Coulter DU 800 spectrophotometer. The concentration of seed fructose was expressed as mg g dwt^−1^.

### *in vivo* nitrate reductase assay

Nitrate reductase activity (NRA) was measured in fully expanded leaves and placed in the buffer solution for *in vivo* NRA assay based on the method of Klepper and Hageman (1969). Briefly, approximately 0.3 g of tissue was placed in 10 mL of potassium phosphate buffer of a concentration of 100 mM, pH 7.5, containing 1% (v/v) 1-propanol, in a flask. The buffer solution with samples was vacuum filtered for 1 min, and then flashed with nitrogen gas for 30 s and then incubated at 30°C. A sample of 0.5 mL was taken from each replicate at regular intervals (0, 60, 120, 180, and 300 min) for nitrite determination. Samples were extracted with 5 mL of deionized water and reacted with 1.0 mL of 1% (w/v) sulfanilamide in 10% v/v HCl and 1.0 mL of *N*-naphthyl-(1)-ethylenediamine dihydrochloride (0.1%). The samples were read after 30 min at 540 nm using a Beckman Coulter DU 800 spectrophotometer. The concentration of nitrite sing KNO_2_ was calculated from a standard calibration curve. Potential NRA (PNRA) under conditions where nitrate is limited was determined by adding exogenous nitrate to the incubation solution at a concentration of 10 mM of KNO_3_. NRA was expressed as NO_2_ g fwt^−1^ h^−1^

### Acetylene reduction assay

Nitrogenase activity (nitrogen fixation, NF) at seed-fill stages (R5–R6) was assayed using the acetylene reduction assay (Hardy et al., 1968; Zablotowicz et al., 1981; Bellaloui and Mengistu, 2008). Roots with nodules intact were excised and incubated in 1 L Mason jars. Three roots were placed in the Mason jars and sealed. A 10% volume of air was removed and replaced with an equal volume of acetylene. Duplicate 1.0 ml gas samples were removed after 1 h of incubation at room temperature and analyzed by gas chromatography (An Agilent HP6960, Agilent Technologies, Wilmington, DE) for ethylene formation. The gas chromatography instrument was equipped with manual injector, injector loop, sample splitter, flame ionization detector (FID), and thermal conductivity detector (TCD). A 0.25 ml sample of gas was directed into a 30 m length × 0.53 mm i.d. alumina megabore column, connected to the FID, and 0.25 ml of sample was injected into a HP- PLOT D column (30 m length × 0.53 mm i.d. megabore with 40 μm film; helium was used as a carrier gas. Chem Station software was used to conduct the integration of chromatographs.

### Leaf water potential measurement

In a non-repeated experiment with three replicates, leaf water potential (LWP, Ψ_w_) was determined on young and fully expanded leaves at vegetative stage using leaf cutter thermocouple psychrometers (J.R.D. Merrill Specialty Equipment, Logan, UT, USA) at mid-day (1200–1300 h). A 5-mm diameter leaf disc was taken and placed in a leaf cutter thermocouple psychrometer. Measurements were conducted on three individual plants (replicate) of each genotype in each water treatment (well-watered and drought stress). The leaf cutter thermocouple psychrometers were placed in a water bath at 25°C for 4 h. Outputs from the psychrometers were recorded by a PSYPRO data logger (WESCOR, Inc., Logan, UT, USA).

### Experimental design and statistical analysis

The experimental design was a randomized complete block design with a split-split plot arrangement of treatments; with watering as a main plot, trait as sub-plot, and genotype as sub-sub-plot. Analysis of variance using Proc Mixed was conducted using a split plot model in SAS (SAS Institute, 2001). Means were separated by Fisher's least significant difference test at the 5% probability level.

## Results

### Analysis of variance

Analysis of variance showed that slow-wilting (Trait), watering treatment (Treat), and their interactions were the main sources of variability for mineral nutrients in leaves. The Trait, Treat and Trait × Treat interaction were significant at *P* ≤ 0.001 for minerals Ca, K, P, B, Cu, Fe, and Na. Trait and Trait × Treat were not significant for S and Mn, and Trait × Treat was not significant for Zn (Table 1). The interaction between Trait and Treat indicated that the responses of these leaf minerals to watering regime (well-watered or drought stressed) were influenced by slow-wilting. The trend was similar for seed protein, oil, fatty acids, and sugars (Table 2), with Trait, Treat, and Trait × Treat interaction being significant for all parameters measured, except Treat was not significant for stearic acid and glucose, Trait was not significant for palmitic acid and Trait × Treat interaction was not significant for linolenic aicd and glucose. This indicates that alteration in seed composition constituents was different among the treatment and trait combinations. For seed minerals, all seed minerals were significantly affected by watering treatment (Table 3). Trait was significantly different for P and B, and the interaction between the Trait and watering treatment was significant only for seed Ca and K. When Trait was replaced by genotype in the model to analyze genotype effects, the results were similar to the analysis of Trait for leaf and seed composition components (data not shown). Since the Trait, Treat, and their interactions were the main significant sources of variability, and the trend of variables was generally similar in both experiments, the results were pooled and combined making the total number of replicates 6.

**Table 1:** **Analysis of variance for responses of leaf minerals, N, S, nitrogen fixation (ARA, μmol of C_2_H_4_ plant^−1^ h^−1^), rate of leaf nitrate assimilation (NRA, μmol NO_2_ g fwt^−1^ h^−1^), and nodule NRA (μmol NO_2_ g fwt^−1^ h^−1^) to slow-wilting phenotype (Trait) under well-watered and drought stressed conditions (Treat) in soybean genotypes differing in slow-wilting trait**.

**Source of variability**	**Df**	**Ca**	**K**	**N**	**P**	**S**	**B**	**Cu**	**Fe**	**Mn**	**Na**	**Zn**	**ARA**	**Leaf NRA**	**Nodule NRA**
Exp	1	NS	NS	***	NS	NS	NS	NS	NS	NS	*	NS	NS	NS	NS
Trait	1	***	***	***	***	NS	***	***	***	NS	***	***	NS	NS	***
Exp × Trait	1	NS	NS	***	NS	NS	NS	NS	NS	NS	NS	NS	NS	NS	NS
Treatment (Treat)	1	***	***	***	***	***	***	***	***	**	***	***	***	***	***
Exp × Treat	1	NS	NS	***	**	*	NS	NS	NS	NS	NS	NS	NS	NS	NS
Trait × Treat	1	***	***	NS	***	NS	**	***	***	NS	***	NS	***	NS	**
Exp × Trait × Treat	1	NS	NS	NS	**	NS	NS	NS	NS	NS	NS	NS	NS	NS	NS

* P ≤ 0.05;

** P ≤ 0.01;

*** p ≤ 0.001. The experiment was repeated twice and number of replicates was six.

**Table 2:** **Analysis of variance for responses of seed composition (protein, oil, fatty acids, and sugars) percentage (%) to slow-wilting phenotype (Trait) under well-watered and drought stressed conditions (Treat) in soybean genotypes differing in slow-wilting trait**.

**Source of variability**	**Protein**	**Oil**	**C16:0**	**C18:0**	**C18:1**	**C18:2**	**C18:3**	**Sucrose**	**Raffinose**	**Stachyose**	**Glucose**	**Fructose**
Exp	NS	NS	NS	NS	NS	NS	**	NS	NS	NS	NS	*
Trait	***	***	***	NS	***	***	***	***	**	***	*	NS
Exp × Trait	NS	NS	NS	***	NS	NS	NS	NS	NS	NS	NS	NS
Treatment (Treat)	***	***	NS	***	***	***	***	***	**	***	NS	***
Exp × Treat	NS	NS	NS	***	**	***	NS	NS	NS	NS	NS	NS
Trait × Treat	***	***	***	**	***	***	NS	**	**	***	NS	***
Exp × Trait × Treat	NS	NS	NS	***	NS	NS	NS	NS	NS	NS	NS	NS

* P ≤ 0.05;

** P ≤ 0.01;

*** p ≤ 0.001. The experiment was repeated twice and number of replicates was six.

**Table 3:** **Analysis of variance for responses of seed minerals and N to slow-wilting phenotype (Trait) under well-watered and drought stressed conditions (Treat) in soybean genotypes differing in slow-wilting trait**.

**Source of variability**	**Seed Ca (%)**	**Seed K (%)**	**Seed N (%)**	**Seed P (%)**	**Seed B (mg kg^−1^)**	**Seed Fe (mg kg^−1^)**
Exp	NS	NS	NS	NS	NS	NS
Trait	NS	NS	NS	*	***	NS
Exp × Trait	NS	NS	NS	NS	NS	NS
Treatment (Treat)	***	***	***	***	***	***
Exp × Treat	NS	NS	NS	NS	NS	NS
Trait × Treat	***	***	NS	NS	*	NS
Exp × Trait × Treat	NS	*	NS	NS	NS	NS

* P ≤ 0.05;

^**^P ≤ 0.01;

*** p ≤ 0.001. The experiment was repeated twice and number of replicates was six.

### Response of leaf nutrients to slow-wilting trait

Under well-watered conditions, genotypes accumulated different concentrations of leaf minerals, (Table 4). The accumulation level of each mineral in leaves depended on the mineral and genotype, but there were trends. Three nutrients that significantly accumulated at higher levels in SLW genotypes were N, B, and Zn (Table 4). Also, Ca, K, Mg, Fe tended to be lower in the SLW lines as compared to checks. Under water stress, however, all leaf minerals, except Mg, S, and Mn had higher concentrations in leaves of SLW genotypes, and the concentration increase between certain SLW genotypes and certain check genotypes reached more than 50% in P, Cu, Fe, and Na. The concentrations of Ca, K, N, and B were also significantly higher in SLW genotypes than the checks, but no clear trends for S, Mn, or Zn concentrations in leaves were observed.

**Table 4:** Responses of leaf minerals, N, and S to slow-wilting (SLW) (drought tolerance) phenotype (Trait) in soybean genotypes differing in slow-wilting trait under well-watered (W) and drought stressed (DS) conditions^*^.

**Genotype**	**Trait**	**Ca (%)**	**K (%)**	**Mg (%)**	**N (%)**	**P (%)**	**S (%)**	**B (mg kg^−1^)**	**Cu (mg kg^−1^)**	**Fe (mg kg^−1^)**	**Mn (mg kg^−1^)**	**Na (mg kg^−1^)**	**Zn (mg kg^−1^)**
**W**													
BOGGS	Check	1.25	2.69	0.63	4.53	0.35	0.24	64.5	4.75	107	112	23.0	79.7
NC-Roy	Check	1.29	3.10	0.63	4.42	0.45	0.26	77.8	4.32	107	83.6	25.5	65.8
NO49646	SLW	1.08	2.19	0.57	5.16	0.44	0.27	82.1	4.42	103	107	25.4	81.0
NTCPR945	SLW	1.13	2.13	0.60	5.40	0.38	0.26	91.5	4.59	94.4	88.3	20.0	90.7
LSD		0.02	0.09	0.01	0.15	0.01	0.004	4.28	0.25	2.16	2.87	1.28	2.11
**DS**													
BOGGS	Check	1.05	2.30	0.70	4.18	0.33	0.26	116	4.63	87.9	127	31.0	92.8
NC-Roy	Check	1.13	2.42	0.69	4.06	0.34	0.31	114	5.67	84.5	102	39.6	92.0
NO49646	SLW	1.42	2.68	0.71	4.84	0.65	0.29	140	7.08	138	121	60.7	93.7
NTCPR945	SLW	1.54	2.82	0.70	4.97	0.64	0.28	161	7.86	127	92.1	73.8	105
LSD		0.035	0.05	0.02	0.26	0.038	0.016	5.76	0.42	3.78	5.08	3.12	3.27

* The experiment was repeated twice and values are means of six replicates.

### Response of seed nutrients to slow-wilting trait

Under well-watered conditions, seed protein was significantly higher in checks than in SLW genotypes, but oil was the opposite (lower), which is consistent with the established inverse relationship between protein and oil (Table 5). Oleic acid level was higher in SLW genotypes than in checks. Both linoleic and linolenic acids were higher in checks than in SLW genotypes. Since the check and SLW genotypes do not have identical genetic background, the differences in protein, oil, and fatty acid levels between checks and SLW genotypes could be due to either the SLW trait, other genotypic differences or both. The range in values for protein, oil, and fatty acids in all genotypes was within the range of normal soybean genotypes. Seed sucrose and stachyose levels were higher in SLW genotypes, and fructose was higher in check genotypes. There was no clear trend in seed raffinose and glucose (Table 4). Under drought stress, seed protein, oleic acid, and sugars were higher in SLW genotypes than checks (Table 5). The most interesting observation was that seed oil was higher in the SLW than the checks under well-watered and drought conditions. Whereas, the protein level went up under dry conditions for both normal and SLW (Table 5). However, linoleic and linolenic acid levels were lower in SLW genotypes. No clear trend for palmitic and stearic acids was evident (Table 5). Under well-watered conditions, Ca and K concentrations in seeds were higher in the checks genotypes than in SLW genotypes, and B concentration was higher in SLW genotypes than in the checks genotypes (Table 6). There were no clear trends for the rest of the nutrients recorded. Under drought stress, Ca, K, and B were higher in SLW genotypes than checks (Table 6). There were no clear trends for N, P, or Fe concentrations observed.

**Table 5:** Responses of seed composition (protein, oil, fatty acids, and sugars) (%) to slow-wilting in soybean genotypes differing in slow-wilting (SLW) trait under well-watered (W) and drought stressed (DS) conditions^*^.

**Genotype**	**Trait**	**Protein**	**Oil**	**C16:0**	**C18:0**	**C18:1**	**C18:2**	**C18:3**	**Sucrose**	**Raffinose**	**Stachyose**	**Glucose**	**Fructose**
**W**													
BOGGS	Check	42.2	19.5	10.5	3.7	20.2	58.5	7.5	3.5	0.47	3.3	2.1	0.94
NC-Roy	Check	41.0	20.1	11.1	3.6	20.0	58.3	6.9	3.6	0.55	3.3	1.7	0.90
NO49646	SLW	40.6	20.8	11.7	3.4	22.2	56.3	6.5	4.3	0.52	3.6	2.2	0.86
NTCPR945	SLW	40.0	21.6	11.7	3.3	22.8	56.3	6.3	3.9	0.52	3.9	1.6	0.70
LSD		0.324	0.18	0.23	0.081	0.47	0.45	0.23	0.17	0.025	0.14	0.19	0.083
**DS**													
BOGGS		43.7	18.8	10.9	3.71	25.4	53.8	6.48	2.28	0.49	4.07	1.23	0.52
NC-Roy		43.7	18.6	11.4	3.72	25.4	53.7	5.88	2.25	0.54	4.85	1.56	0.51
NO49646		42.7	20.6	11.9	3.72	29.7	48.1	5.97	3.20	0.60	6.62	1.93	0.69
NTCPR945		42.8	20.5	11.1	3.88	33.4	45.9	5.60	4.20	0.70	6.27	1.88	0.73
LSD		0.35	0.33	0.27	0.20	0.50	0.42	0.16	0.15	0.027	0.35	0.18	0.026

* C16:0, palmitic acid; C18:0, stearic acid; C18:1, oleic acid; C18:2, linoleic acid; C18:3, linolenic acid. The experiment was repeated twice and values are means of six replicates.

**Table 6:** Responses of seed minerals and N to slow-wilting in soybean genotypes differing in slow-wilting (SLW) trait under well-watered (W) and drought stressed (DS) conditions^*^.

**Genotypes**	**Trait**	**Ca (%)**	**K (%)**	**N (%)**	**P (%)**	**B **(mg kg^−1^)****	**Fe **(mg kg^−1^)****
**W**							
BOGGS	Check	0.39	1.69	5.25	0.49	36.5	109
NC-Roy	Check	0.45	1.48	5.45	0.54	44.5	96.5
NO49646	SLW	0.35	1.26	4.93	0.55	60.9	89.7
NTCPR945	SLW	0.33	1.42	5.32	0.62	67.7	93
LSD		0.019	0.043	0.216	0.026	6.24	9.82
**DS**							
BOGGS		0.23	1.22	3.91	0.39	31.3	61.0
NC-Roy		0.29	1.32	4.07	0.36	34.8	59.2
NO49646		0.31	1.37	3.68	0.35	47.5	58.5
NTCPR945		0.37	1.41	4.15	0.30	45.5	53.8
LSD		0.020	0.027	0.23	0.028	2.48	2.14

* The experiment was repeated twice and values are means of six replicates.

### Nitrogen fixation, assimilation, and leaf water potential

**Figure 1:**
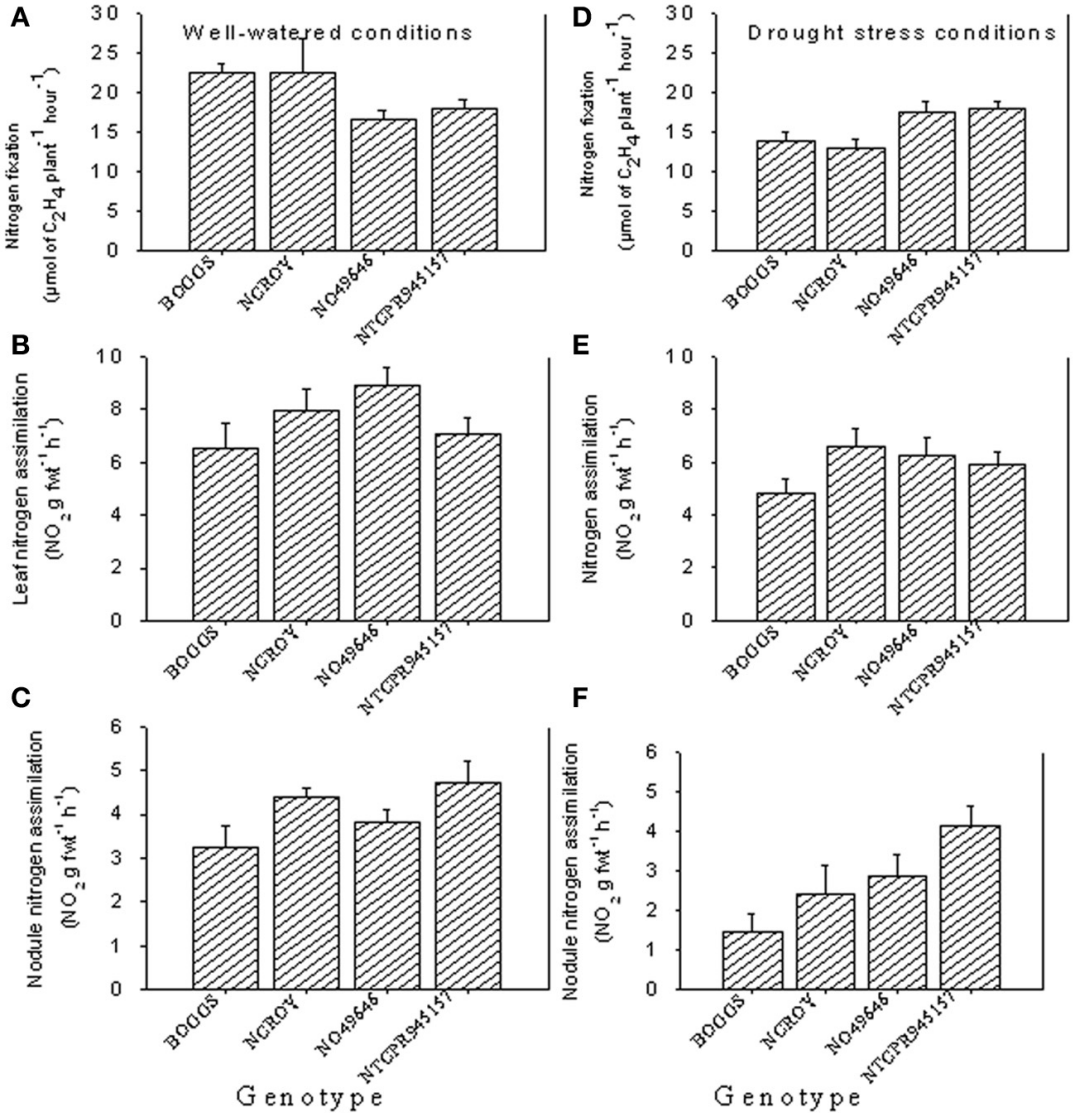
**Responses of nitrogen fixation and leaf and nodule nitrogen assimilation under well-watered conditions (A–C) and drought stress conditions (D–F) in soybean genotypes differing in slow-wilting (SLW) phenotype**.

**Figure 2:**
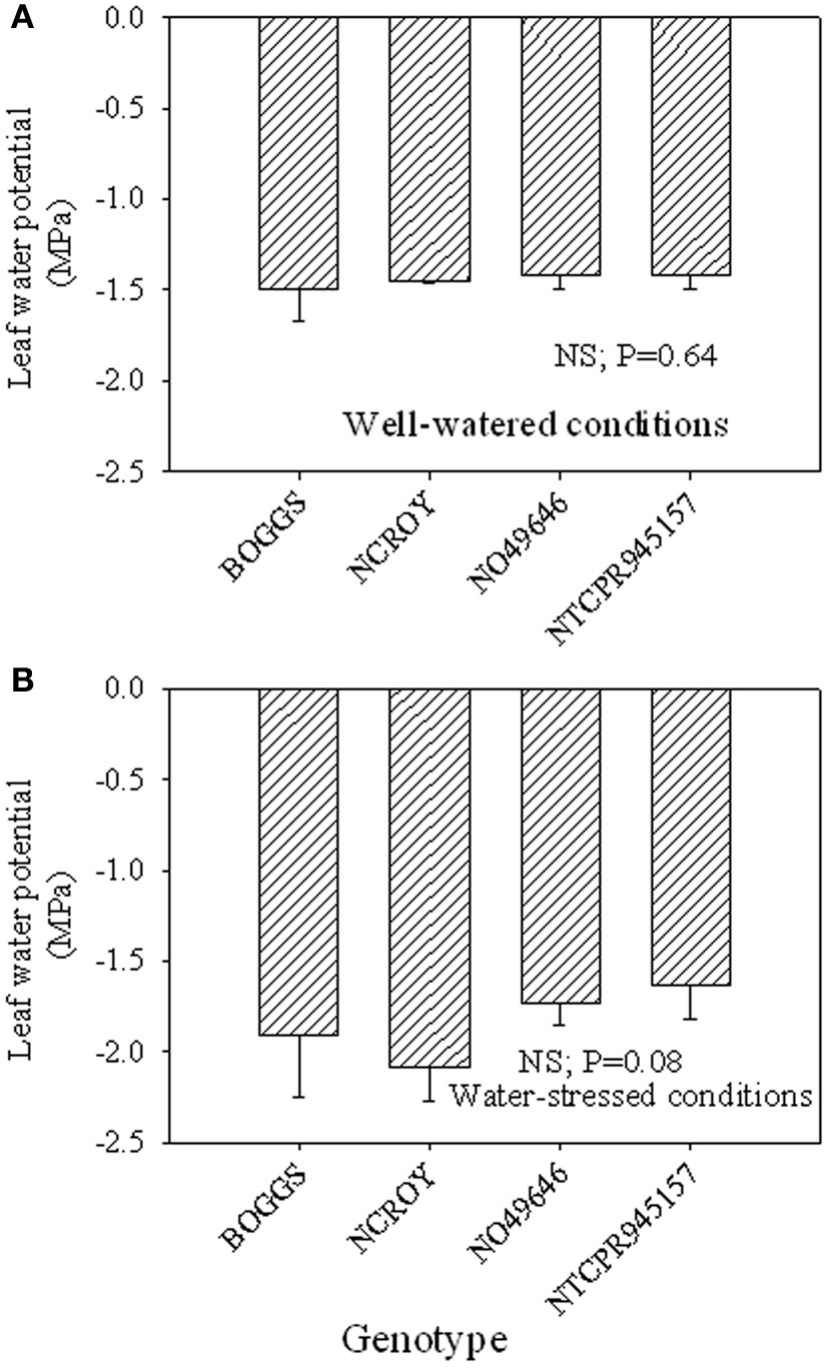
**Responses of leaf water potential to well-watered conditions (A) and drought stress conditions (B) in soybean genotypes differing in slow-wilting (SLW) phenotype**.

**Figure 3:**
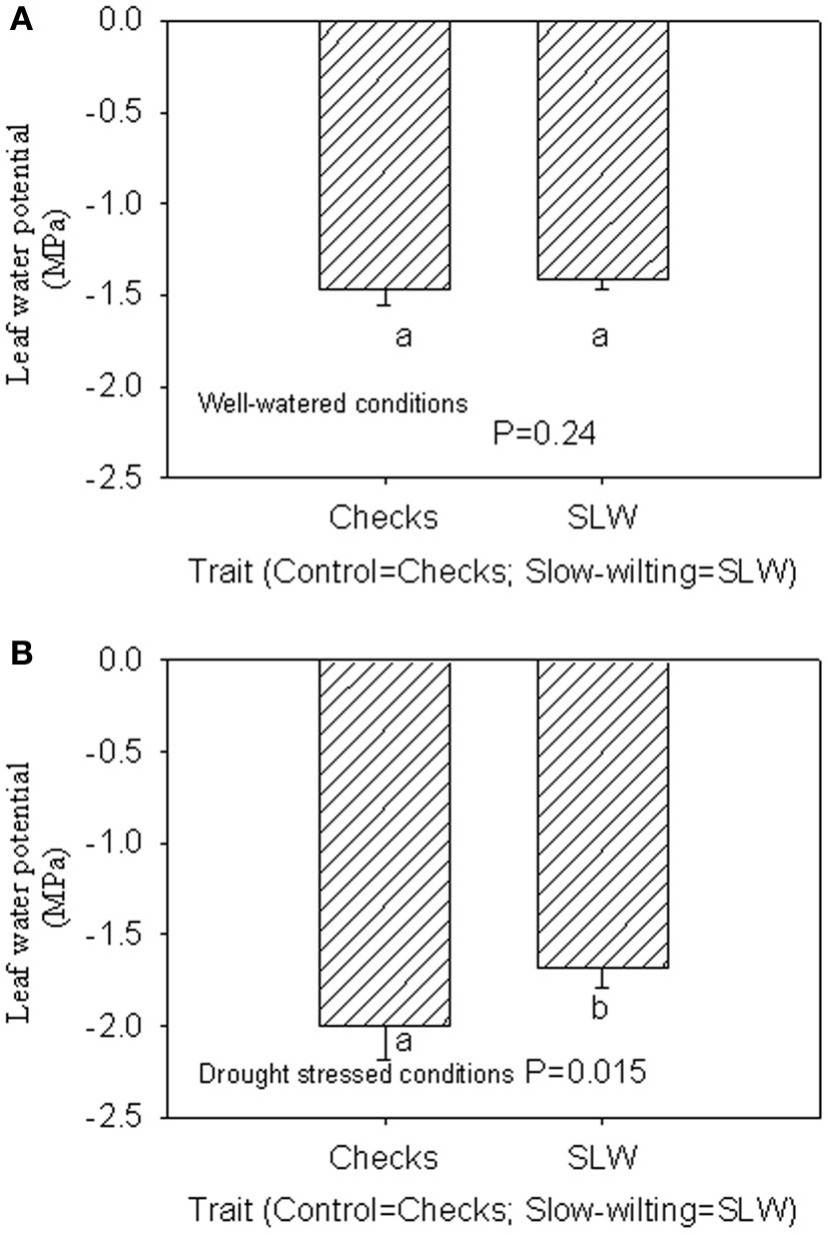
**Responses of leaf water potential to well-watered conditions (A) and drought stress conditions (B) across checks and slow-wiling (SLW) soybean genotypes**.

Under well-watered conditions, nitrogen fixation was lower in SLW than checks; however, leaf nitrogen assimilation was highest in NO49646 (SLW) and lowest in Boggs (check) Figures 1A,B). Nodule nitrogen assimilation was highest in NTCPR945157 (SLW) and lowest in Boggs (Figure 1C). However there were no consistent differences between the SLW or checks for either leaf or nodule nitrogen assimilation. Under drought stress (Figures 1D–F), both SLW genotypes (NO49646 and NTCPR945157) showed higher nitrogen fixation rates than checks. Comparing the well-watered to the drought stress treatments, ARA in SLW genotypes did not significantly change, but leaf NRA decreased, and nodule NRA was not consistent (Figures 1A–F). Nitrogen assimilation in leaves or nodules did not show any consistent significant differences between the SLW and checks. Leaf water potential (LWP) was not significantly different among genotypes under well-watered conditions or drought stress (Figures 2A,B), may be due to genotypic differences and not the trait. However, when the analysis was done across the two checks and the two SLW genotypes, leaf water potential in the checks was significantly (*P* = 0.015) lower (−2.00 MPa) than in SLW genotypes (−1.68 MPa) under drought stress conditions (Figures 3A,B).

## Discussion

### Response of leaf nutrients to slow-wilting trait

The higher concentrations of Ca, K, Fe in the checks than SLW genotypes could be due to the inherited genotypic differences. A significant crossover interaction is evident for Ca, K, N, Fe among the two treatments. For these minerals accumulation, the SLW were lower than the checks under well watered conditions, but under drought stress the SLW had higher concentrations than the checks. For P, there was no evident difference between SLW and the checks under well-watered conditions but the magnitude of response to drought stress between the genotypes was not the same. The P, Cu, and B concentrations in the SLW lines increased under drought stress more than in the checks. The higher concentrations of Ca, K, P, B, Cu, Fe, and Na in leaves of SLW genotypes compared to the check genotypes under drought stress may indicate the involvement of these nutrients in drought stress response directly or indirectly. Previous research showed that higher accumulation of K (Bartels and Sunkar, 2005), Ca, Cl, and Na in leaves under drought stress (Knight et al., 1997; Schroeder et al., 2001; Furlan et al., 2012) increased osmotic pressure and regulated stomatal opening (Bartels and Sunkar, 2005) in order to maintain cell membrane integrity and function (Wei et al., 2003).

Potassium plays a major role under drought stress because of its involvement in protein synthesis, glycolytic enzymes, photosynthesis, cell expansion, and turgor, carbohydrate movement, stomatal regulation, osmoregulation, energy status, charge balance, homeostasis (Hsiao, 1973; Marschner, 2012), and transpiration (Andersen et al., 1992). Osmotic adjustment using ions such as K increased drought tolerance because it is more energy efficient for plants to cope with drought using K than to produce organic solutes (Hsiao, 1973; Hu and Schmidhalter, 2005). For example, Morgan (1992) investigated the genotypic differences in osmoregulation under water stress condition in a glasshouse using sap samples from expanded wheat flag leaves of breeding lines with high and low osmoregulation, and found that high osmoregulation was due to potassium accumulation (78%) and amino acids (22%). Ma et al. (2004), working on the accumulation and osmotic adjustment in leaves of *Brassica* oilseeds under glasshouse conditions, found that the osmotic adjustment in expanded leaves was due to nitrate (42–47%), soluble sugars (31–38%), and proline (11–14%). In expanding leaves, however, the osmotic adjustment was due to K accumulation (23–27%), proline (17–22%), and less due to nitrate and soluble sugars, indicating the complexity of K dynamics due to its fast mobility in the phloem. Generally, K availability under drought stress decreased due to a decrease in K mobility (Hu and Schmidhalter, 2005), and under these conditions, application of foliar K was found to decrease the adverse effects of drought (Andersen et al., 1992; Sangakkara et al., 2001). In our experiment and under drought stress, K level in leaf tissues of SLW genotypes was higher than the check genotypes, and maintaining high level of K in leaves resulted in higher seed K in SLW genotypes, suggesting this is a possible mechanism to maintain adequate K concentration in seeds under drought stress. Under severe drought (soil water potential = −199 kPa), however, our preliminary results showed that foliar K application at a rate of 1.75% as K_2_SO_4_ to SLW genotypes resulted in an increase of K in leaves, but very low level of K in seeds was observed in all genotypes, suggesting the response of K to slow wilting may depend on the severity of drought.

Phosphorus concentrations in leaves of SLW genotypes were higher than in the check genotypes, suggesting that the SLW genotypes had the ability to maintain higher P concentrations in leaves under drought stress. Phosphorus is involved in several physiological and biochemical processes such as energy storage and transfer, photosynthesis, and enzyme regulation, and is also integral part of nucleic acids, phospholipids, and phosphoproteins (Hu and Schmidhalter, 2005; Marschner, 2012). It was reported that drought stress inhibits P absorption, uptake, translocation, and accumulation, although the degree of influence of drought on P is dependent on the severity of drought stress (Rasnick, 1970; Pinkerton and Simpson, 1986). The role of P under drought stress was attributed to increases in water-use efficiency, stomatal conductance (Brück et al., 2000), photosynthesis (Ackerson, 1985), higher cell membrane stability, and water relations (Sawwan et al., 2000). It was found that P fertilizer application under drought stress can improve plant growth (Ackerson, 1985; Garg et al., 2004; Hu and Schmidhalter, 2005). In our experiment and under drought stress, P in leaves was higher in SLW genotypes (increase of 94% in NTCPR94-5157 and 91% in N04-9646) compared with their equivalent checks Boggs and NC-Roy, respectively), showing a positive response to the SLW trait. This increase in P in leaves was not reflected in P in seeds, perhaps due to low mobility and translocation of P from leaves to seed.

Calcium levels in leaves and seeds were higher in SLW genotypes than in check genotypes, but this response was only shown under drought stress, and not under well-watered conditions where Ca in leaves and seeds were higher in the check genotypes than in SLW genotypes. Calcium is thought to have a role in drought stress because of its involvement in water and solute movement, stomatal function, and signaling systems under biotic and abiotic stress (McLaughlin and Wimmer, 1999), osmoregulation under drought stress (Knight et al., 1997; Bartels and Sunkar, 2005), and Ca^2+^-ATPases to restore and maintain homeostasis by pumping Ca^++^out of the cytosol to terminate a signaling event (Sze et al., 2000). The decrease of Ca in leaves and seeds in the checks may be due to the decrease of Ca uptake and lack of ability of these genotypes to maintain high levels of Ca in leaves and seeds. Compared with the checks, Ca in SLW genotypes was higher under drought stress (increase of 47% in NTCPR94-5157 and 26% in N04-9646 compared with their equivalent checks Boggs and NC-Roy, respectively). The higher increase of Na, B, Fe, Cu in SLW genotypes may indicate possible involvement of these nutrients in stomatal opening and osmoregulation. The little or no response of Mg and S could indicate minimal role of these nutrients in drought tolerance and osmoregulation. Although information available on the effect of drought on S and Mg nutrition of plants is limited (Hu and Schmidhalter, 2005), it was assumed that drought decreases the uptake of Mg and S (Scherer, 2001). Hu and Schmidhalter (2005) reported that B deficiency occurs under drought due to low mineralization that dissociates B from organic matter and induces Mn, Mo, Fe, and Zn deficiencies.

### Response of seed nutrients to slow-wilting trait

The seed protein, oil, and fatty acid levels in the genotypes were all within normal levels found in soybean lines. The higher seed protein and different fatty acids composition of seed under well-watered conditions in genotypes could be due to genotypic differences. Usually, when soybeans are grown under different conditions, it is observed that when protein percentage increases in a line, the oil percentage will decrease. N049646 was notable in that the oil percentage decreased by only 1% when protein percentage protein percentage increased by 5% between well-watered and drought stress conditions (Table 7). In the other genotypes, protein increased from 3.6 to 7.0% and oil decreased by 3.6 to 7.5% (Table 7). Under drought stress, protein and oleic acid increased and linoleic and linolenic acids decreased in all genotypes, but the changes were more extreme in the SLW genotypes for protein and oleic acids. The higher oleic acid in SLW genotypes suggests a possible role of oleic acid as a drought stress indicator. It must be noted that the check genotypes under drought stress conditions also exhibited high protein and oleic acid compared with those grown under well-watered conditions, but the rate of increase was lower than in SLW genotypes.

**Table 7:** **Percentages increase in seed composition (%) in response to slow-wilting under well-watered (W) and drought stressed (DS) conditions in soybean Boggs and NC-Roy (checks) and NO49646 and NTCPR945 (slow-wilting) genotypes**.

**Constituent**	**W**	**DS**	**delta**	**% of W**	**W**	**DS**	**delta**	**% of W**
	**Boggs**				**NC-Roy**			
Protein	42.2	43.7	1.5	3.6	41	43.7	2.7	6.6
Oil	19.5	18.8	−0.7	−3.6	20.1	18.6	−1.5	−7.5
C18:1	20.2	25.4	5.2	25.7	20	25.4	5.4	27.0
C18:3	7.5	6.48	−1.02	−13.6	6.9	5.88	−1.02	−14.8
Sucrose	3.5	2.28	−1.22	−34.9	3.6	2.25	−1.35	−37.5
Raffinose	0.47	0.49	0.02	4.3	0.55	0.54	−0.01	−1.8
Stachyose	3.3	4.07	0.77	23.3	3.3	4.85	1.55	47.0
Glucose	2.1	1.23	−0.87	−41.4	1.7	1.56	−0.14	−8.2
Fructose	0.94	0.52	−0.42	−44.7	0.9	0.51	−0.39	−43.3
	**NO49646**				**NTCPR945**			
Protein	40.6	42.7	2.1	5.2	40	42.8	2.8	7.0
Oil	20.8	20.6	−0.2	−1.0	21.6	20.5	−1.1	−5.1
C18:1	22.2	29.7	7.5	33.8	22.8	33.4	10.6	46.5
C18:3	6.5	5.97	−0.53	−8.2	6.3	5.6	−0.7	−11.1
Sucrose	4.3	3.2	−1.1	−25.6	3.9	4.2	0.3	7.7
Raffinose	0.52	0.6	0.08	15.4	0.52	0.7	0.18	34.6
Stachyose	3.6	6.62	3.02	83.9	3.9	6.27	2.37	60.8
Glucose	2.2	1.93	−0.27	−12.3	1.6	1.88	0.28	17.5
Fructose	0.86	0.69	−0.17	−19.8	0.7	0.73	0.03	4.3

The experiment was repeated twice and values are means of six replicates.

A positive response of seed sugars, Ca, K, and B to drought stress in SLW genotypes was observed, indicating that sugars, especially sucrose, raffinose, and stachyose may have contributed to drought tolerance and may be considered as drought stress indicator for the SLW trait. In our experiments, the percentage increases in seed sucrose, raffinose, stachyose, glucose, and fructose in NTCPR945 under drought stress were 7.7, 34.6, 60.8, 17.5, 4.3%, respectively, although the percentages increase in NO49646 were less than in NTCPR946 (Table 7)The biological function of raffinose and stachyose are not well understood (Ren et al., 2009), although the oligosaccharides (sucrose, raffinose, and stachyose) involvement in seed quality (Wilson, 2004), desiccation tolerance during seed development and maturation, and protection of seeds against drought damage were previously reported. For example, it was reported that the accumulation of compatible solutes such as sugars (non-structural sugars), including sucrose, raffinose, stachyose (Chen and Murata, 2002), and sucrose, hexoses, and sugar alcohols accumulation are related to osmotic stress tolerance (Streeter et al., 2001; Taji et al., 2002). It was explained that the accumulation of sugars was a result of starch hydrolysis and sugars conversion (Ingram et al., 1997), where sugars act as osmotica to protect specific macromolecules and stabilize membrane structures, protect cells during desiccation (Phillips et al., 2002), and prevent membrane fusion. In our experiment, the accumulation of raffinose oligosaccharides (RFOs) was higher in SLW genotypes than in check genotypes under drought stress, and this is in agreement with other reports that many seeds accumulate considerable amounts of RFOs such as raffinose and stachyose under drought stress. For example, the high accumulation of RFOs such as raffinose and stachyose were thought to play a role in the acquisition of desiccation tolerance, and overexpression of galactinol synthase (catalyzes the first step in the biosynthesis of RFOs) led to higher accumulation of galactinol and raffinose and improved drought tolerance (Taji et al., 2002). In spite of the existing knowledge about RFOs, the mechanisms of how these sugars are involved in drought tolerance are still not fully understood (Chen and Murata, 2002; Bartels and Sunkar, 2005).

The higher accumulation of compatible solutes such as sucrose, hexoses, mannitol, sorbitol, and isotol (Gilmour et al., 2000; Streeter et al., 2001; Taji et al., 2002) and amino acids such as proline, glycine, and betaine to maintain turgor and stabilize proteins and cell structures (Yancey et al., 1982; Burg et al., 1996) were previously reported. The ability to maintain higher Ca and K in seeds in SLW genotypes than the checks may indicate that both Ca and K constitute the main cations in homeostasis regulation under drought stress, and since K is a quite mobile cation (Marschner, 2012), K movement from leaves to seed occurred under drought stress. The decrease of K in checks in DS compared with W treatment may be due to inability of these genotypes to maintain K concentration in their seed probably due to due lack of homeostasis regulation and water conservation. This explanation could partially be supported by the lower water potential observed in the check phenotypes compared with the SWL genotypes under drought, resulting in possible reduction in K uptake and movement of K from leaves to seed.

Under severe stress (soil water potential −199 kPa), K in leaves and seed was significantly lower than under moderate drought stress (data not shown), indicating that the mobility can only occur up to a certain drought stress level. Since both K and B had clear responses (higher accumulations in SLW genotypes than the checks under drought stress), to further investigate their dynamics in leaves and seed under severe drought, foliar K and B were applied to SLW genotypes only. Foliar K at a rate of 1.75% as K_2_SO_4_ to SLW genotypes resulted in higher K in leaves and seeds under moderate drought stress (−90 to −100 kPa), but only a small increase in leaves and no significant increase in seeds under severe drought stress (−199 KPa) (data not shown). Foliar application of B to SLW genotypes at a rate of 1.1 kg ha^−1^ as H_3_BO_3_ resulted in higher B in leaves and seed and higher glucose, fructose, and sucrose under well-watered and moderate drought stress conditions, but not in severe drought stress (data not shown). It must be noted that the rate of increase of B during R5–R6 in leaves and seeds, which resulted from foliar B application, was lower than that of K increases, perhaps due to limited mobility of B in the soybean phloem compared with K, which is quite mobile. Foliar B application under severe drought stress did not result in higher B in seeds of SLW genotypes, suggesting that B movement of B from leaves to seed under drought stress is restricted even when foliar B is applied.

### Nitrogen fixation and assimilation, and leaf water potential

The differences between genotypes for nitrogen fixation and leaf nitrogen assimilation under well watered conditions could be due to genotypic differences rather than SLW trait, as the SLW trait may be expressed only under drought stress conditions. The lower rates of nitrogen fixation and nodule nitrogen assimilation under drought conditions in checks compared with SLW genotypes could be due to drought stress effects and lower nutrient uptakes as a result, especially K, B, Fe, Cu, and Zn that are indirectly affect nitrogen fixation. Also, the check genotypes may lack the ability for homeostasis regulation and water conservation, resulting in inhibition of nitrogen fixation and nodule nitrogen assimilation compared with the SLW genotypes. The higher nitrogen fixation and nodule nitrogen assimilation in SLW genotypes than the check genotypes suggested that nitrogen fixation and nodule nitrogen assimilation are more indicative in drought stress than is leaf nitrogen assimilation. The inhibition of nitrogen fixation and nitrogen assimilation under drought stress is due to the negative effects of drought on the activity of nitrogenase and nitrate reductase, although nitrogenase is more sensitive than nitrate reductase (Purcell and King, 1996). Previous research reported that mechanisms involved in the physiological response of nitrogen fixation to drought included carbon shortage, nodule carbon metabolism, oxygen limitation, and feedback of nitrogen fixation products (Serraj et al., 1999; Serraj, 2003) such as amides (mainly asparagine) and ureides (allantoin and allantoic acid) (Schubert et al., 1995).

The higher leaf water potential in the SLW genotypes compared with the check genotypes may indicate that leaf water potential could be involved in the SLW trait as leaf water potential may be related to stomatal conductance's role to restrict water loss and conserve water, supporting previous research. For example, crop response to water deficit involves physiological changes to minimize water loss such as closing stomata and reducing leaf surface area (O'Toole and Moya, 1978) and canopy wilting (Lawlar and Cornic, 2002). However, canopy wilting has not been well studied (Lawlar and Cornic, 2002; King et al., 2009), and previous research showed that soybean genotypes differ in canopy wilting under water deficit (Sloane et al., 1990).

### Response mechanisms of slow-wilting trait to drought tolerance

When the mechanisms of SLW trait were evaluated, it was suggested that drought tolerant genotype had higher soil moisture during drought compared with a fast-wilting genotype due to low rates of transpiration and decline in water use in the whole plant during soil water deficit (Sloane et al., 1990; Fletcher et al., 2007; King et al., 2009; Sadok and Sinclair, 2009). The ability to conserve soil moisture by lowering transpiration rate in SLW genotypes was to maintain turgor pressure compared with fast-wilting genotypes Fletcher et al., 2007; Charlson et al., 2009; King et al., 2009; Ries et al., 2012. Hufstetler et al. (2007) investigated three physiological traits (water use efficiency, decline of water use in the whole plant during drought stress, and low leaf epidermal conductance). They conducted two greenhouse experiments and compared these three traits in six soybean plant introductions, eight breeding lines, and nine cultivars during vegetative growth. During these experiments, the water use efficiency (ratio of total plant dry weight to total water used), low leaf epidermal conductance, and the critical soil water content at which each plant began to reduce its water use were determined. They found significant variation among the tested genotypes for the three traits. They found a negative correlation between water use efficiency and leaf epidermal conductance across genotypes, and adapted genotypes had higher water use efficiency and lower leaf epidermal conductance. However, PI 471938 and its progeny N98-7264 did not follow the same trend.

King et al. (2009) studied the slow-wilting trait in a two-years field experiment in 19 soybean genotypes among which PI 416937 and PI 471938 were among the slowest wilting genotypes, and breeding lines 93705-34 and 93705-95 were among the fastest wilting. They found in a separate field experiment that volumetric soil water content was higher in slow-wilting PI 416937 than fast-wilting 93705-95; however, wilting in both genotypes responded similarly to soil water content. They also studied the SLW trait under growth chamber conditions and found that transpiration was reduced due to drought stress similarly for slow- and fast-wilting genotypes, and wilting response to soil water was the same for slow-wilting PI 416937 and fast-wilting genotypes 93705-34 and A5959. Slow-wilting 93705-36 exhibited wilting at lower soil water content than PI416937, 93705-34, and A5959. King et al. (2009) concluded that more than one mechanism may be involved in slow wilting. Also, Sloane et al. (1990), using a slow-wilting genotype PI 416937 and fast-wilting cultivar Forrest, found that water stress reduced leaf water potential equally for both genotypes, but PI 416937 maintained lower levels of solute potential and higher pressure potential and relative water content than Forrest. They suggested that under water stress PI 416937 may accumulate more solutes in leaves than Forrest, and concluded that PI 416937 may be an important source of drought tolerance for breeding programs.

## Conclusion

The physiological mechanisms explaining the response of the slow-wilting trait to drought stress are complex (Charlson et al., 2009), still not well understood (King et al., 2009), and more than one mechanism could be involved (Hufstetler et al., 2007). The higher accumulation of specific minerals such as K, Ca, B, Na, organic compounds such as sucrose, raffinose and stachyose, and oleic acid under drought stress in SLW genotypes could be one of the mechanisms used to maintain cell turgor, conserve water, and achieve osmoregulation under drought stress. The contribution of S, and micro-nutrients such as B, Mn, Cu, and Zn is still not known, and could be important as these minerals are involved in several enzymatic physiological and biochemical processes. Nitrogen metabolism, especially nitrogen fixation is more sensitive to drought than nitrogen assimilation, supporting previous findings. Leaf water potential could be used as a drought tolerance indicator in slow-wilting genotype selection as slow wilting genotypes exhibited higher leaf water potential than their equivalent checks. Higher leaf water potential in SLW genotypes could indicate that the SWL genotypes conserve water by maintaining more water in the leaves, and maintaining adequate leaf and seed nutrients, and achieve homeostasis. Leaf and seed nutrients exhibited higher accumulation in SLW genotypes, and this knowledge could help soybean breeders to select for drought tolerance.

### Conflict of interest statement

The authors declare that the research was conducted in the absence of any commercial or financial relationships that could be construed as a potential conflict of interest.
